# PSAT1 mediated EMT of colorectal cancer cells by regulating Pl3K/AKT signaling pathway

**DOI:** 10.7150/jca.93789

**Published:** 2024-04-15

**Authors:** Mingjin Wang, Houshun Zhang, Zhiyuan Lu, Wenrui Su, Yanan Tan, Jiayu Wang, Xiaoyi Jia

**Affiliations:** 1 School of Pharmacy, Anhui University of Traditional Chinese Medicine, 230012 Hefei, Anhui, China.; 2 The Key Laboratory of Hepatobiliary Pancreas, Southern District, Anhui Provincial Hospital, The First Affliated Hosnital of USTC, University of Science and Technology of China, 230022 Hefei, Anhui, China.; 3 Department of Pathology, Anhui Provincial Hospital, The First Affliated Hosnital of USTC, University of Science and Technology of China, 230002 Hefei, Anhui, China.

**Keywords:** PSAT1, Colorectal cancer, EMT, PI3K/AKT pathway, LY294002

## Abstract

**Background:** The metastasis of colorectal cancer (CRC) is one of the significant barriers impeding its treated consequence and bring about high mortality, less surgical resection rate and poor prognosis of CRC patients. PSAT1 is an enzyme involved in serine biosynthesis. The studies showed that PSAT1 plays the part of a crucial character in the regulation of tumor metastasis. And Epithelial-Mesenchymal Transition (EMT) is a process of cell reprogramming in which epithelialcells obtain mesenchymal phenotypes. It is a crucial course in promoting cell metastasis and the progression of malignant tumors. The relationship between PSAT1 and EMT in colorectal cancer, as well as the underlying molecular mechanisms, remains enigmatic and warrants thorough exploration. These findings suggest that PSAT1 may serve as a promising therapeutic target for mitigating colorectal cancer metastasis and holds the potential to emerge as a valuable prognostic biomarker in forthcoming research endeavors.

**Materials and Methods:** Utilizing TCGA dataset in conjunction with clinical CRC specimens, our initial focus was directed towards an in-depth examination of PSAT1 expression within CRC, specifically exploring its potential correlation with the adverse prognostic outcomes experienced by patients. Furthermore, we conducted a comprehensive investigation into the regulatory influence exerted by PSAT1 on CRC through the utilization of siRNA knockdown techniques. In the realm of *in vitro* experimentation, we meticulously evaluated the impact of PSAT1 on various facets of CRC progression, including cell migration, invasion, proliferation, and colony formation. In order to elucidate the intricate effects in question, we adopted a multifaceted methodology that encompassed a range of assays and analyses. These included wound healing assays, transwell assays, utilization of the Cell Counting Kit-8 (CCK-8) assay, and colony formation assays. By employing this diverse array of investigative techniques, we were able to achieve a comprehensive comprehension of the multifaceted role that PSAT1 plays in the pathogenesis of colorectal cancer. This multifarious analysis greatly contributed to our in-depth understanding of the complex mechanisms at play in colorectal cancer pathogenesis. Using WB and PCR experiments, we found that PSAT1 has a role in regulating EMT development in CRC.In terms of mechanism, we found that PSAT1 affected EMT by Regulating Pl3K/AKT Signaling Pathway.

**Results:** Our investigation revealed a noteworthy down-regulation of PSAT1 expression in CRC specimens. Importantly, this down-regulation exhibited a significant positive correlation with the unfavorable prognosis of patients afflicted with CRC. Functionally, our study showcased that the siRNA-mediated knockdown of PSAT1 markedly enhanced various key aspects of CRC pathogenesis in an *in vitro* setting. Specifically, this included a substantial promotion of CRC cell migration, invasion, proliferation, and colony formation. Moreover, the silencing of PSAT1 also demonstrated a substantial promotion of the EMT process. Intriguingly, our research unveiled a hitherto unexplored mechanism underlying the regulatory role of PSAT1 in CRC and EMT. We have established, for the first time, that PSAT1 exerts its influence by modulating the activation of the PI3K/AKT Signaling Pathway. This mechanistic insight provides a valuable contribution to the understanding of the molecular underpinnings of CRC progression and EMT induction mediated by PSAT1.

**Conclusions:** In unison, our research findings shed light on the previously uncharted and significant role of the PSAT1/PI3K/AKT axis in the initiation of the EMT process in CRC. Furthermore, our discoveries introduce a novel biomarker with potential implications for the clinical diagnosis and treatment of CRC.

## Introduction

Colorectal cancer (CRC) stands as a highly prevalent malignancy on a global scale, ranking as the third most substantial contributor to the heightened burden of cancer-related morbidity and mortality among human populations 1. Notably, within the context of China, the incidence of colorectal cancer has surged to second place, paralleled by a commensurate elevation in its mortality rate, now occupying the fourth position. This epidemiological trend has cast a profound shadow over the quality of life and overall health of the Chinese populace 2. It is well-established within the medical literature that one of the primary drivers behind the mortality associated with CRC pertains to its propensity for distant invasion and metastasis 3. Metastasis and distant invasion stand as the predominant culprits behind the mortality rates in nearly 90% of colorectal cancer cases. Despite substantial progress in clinical diagnostic methods and the advent of comprehensive treatment modalities, the prognosis for individuals afflicted with metastatic CRC remains grim, marked by elevated mortality rates 4,5. All in all,to enhance the prognosis of colorectal cancer treatment, it is essential to identify the underlying mechanism of metastasis and studying how to inhibit distant metastasis of colorectal cancer is become crucial.

PSAT1, an enzymatic entity tasked with overseeing the conversion of phosphoserine into serine, has garnered considerable interest among the scientific community. This heightened attention is primarily attributed to its notable presence in a diverse array of tumor types 6-10. Multiple studies have shown that changes in expression of PSAT1 is correlated with malignant metastasis, chemotherapy susceptibility, and poor prognosis 11. The PSAT1 plays a key element in the occurrence and development of a variety of tumors, and therefore it is a key point in the study of cancer development 12-16. The upregulation of PSAT1 has been observed to stimulate the proliferation of estrogen receptor-negative breast cancer cells, while its overexpression has been shown to enhance the metastatic potential of lung adenocarcinoma, as indicated by prior investigations 17-19. Additionally, it is noteworthy that PSAT1 has been identified as a valuable prognostic indicator in the context of low-grade gliomas 20. Furthermore, there exists a significant association between PSAT1 expression and the growth and prognosis of epithelial ovarian cancer 21. However, since the mechanism of action of PSAT1 in CRC is still unclear, it is important to study the development of PSAT1 in colorectal cancer cells.

Epithelial-to-mesenchymal transition (EMT) refers to a special cellular process in which cells acquire mesenchymal characteristics when they lose epithelial properties 22,23. EMT plays a pivotal role in tumour genesis and metastasis 24. In the in-depth study of EMT, epithelial tumor cells go through significant morphological and phenotypic changes, including loss of tight junction function between cells, changes in cell polarity, and rearrangement of the cytoskeleton, which make the cells more invasive and accelerate a malignant phenotype 25,26. EMT contributes to the progression of various cancers, making tumor cells aggressive and leading to metastatic spread to distant organs 27. E-cadherin mediates intercellular adhesion, a property that is lost during carcinogenesis. One of the most noteworthy characteristics contributing to the onset of EMT was the reduction in the overall expression of E-cadherin, accompanied by a concurrent elevation in the global expression levels of N-cadherin and α-SMA 28-30. In conclusion, this study will further explore the mechanism related to PSAT1 regulation of EMT process in CRC cells.

The phenomenon of EMT constitutes a multifaceted and intricately regulated process, characterized by numerous sequential steps and governed by an array of intricate regulatory mechanisms 31. Among the signaling pathways implicated in orchestrating EMT, noteworthy candidates encompass the MAPK/ERK, Wnt/β-catenin, and PI3K/AKT/GSK-3β pathways 32. Of particular significance, the PI3K/AKT signaling pathway has garnered substantial attention for its continuous modulation of EMT activation throughout the progression of various cancers. Notably, this pathway plays a pivotal role in an assortment of cellular processes, including glucose metabolism, apoptosis, cell proliferation, and cell migration 33-35. Recent investigations have underscored the multifaceted interplay between the PI3K/AKT signaling pathway and the development of EMT in cancer, thereby influencing tumor invasion and metastasis 36-38. Exemplifying this intricate relationship, prior research has unveiled the role of cPLA2α as a mediator of the EMT process via its influence on the PI3K/AKT pathway 39. Moreover, compelling evidence suggests that the PI3K/AKT signaling pathway exerts a regulatory role in modulating TGF-β-induced EMT 40. In the context of bile duct carcinoma, the activation of EMT is facilitated through the PLCB1-PI3K-AKT signaling axis 41. Consequently, the regulatory influence of the PI3K/AKT signaling pathway on EMT holds paramount importance, rendering it a promising target for the prevention, diagnosis, and treatment of malignant metastatic tumor development, garnering widespread attention within the scientific community 42,43.

In the course of our investigation, we delved into the expression patterns and functional roles of PSAT1 in CRC, as well as the underlying mechanisms governing its impact on CRC cellular biology. Our research has unveiled a notable down-regulation of PSAT1 in both CRC cell lines and tumor specimens obtained from CRC patients. Furthermore, we have established a positive correlation between the expression levels of PSAT1 and the overall survival rates among individuals afflicted by colorectal cancer. Moreover, our study has brought to light the influence of PSAT1 on the invasive and metastatic capacities of CRC cells. In the realm of mechanistic inquiries, it has been discerned that PSAT1 exerts its effects on the EMT process through modulation of the PI3K/AKT signaling pathway. The outcomes of this investigation underscore the potential of PSAT1 as not only a valuable prognostic biomarker but also as a promising therapeutic target for the management of metastatic colorectal cancer.

## Materials and Methods

### Cell culture

The NCM460 colon epithelial cell line, as well as the CRC cell lines SW620 and DLD-1, were acquired from the Cell Repository of the Chinese Academy of Sciences in Shanghai. Furthermore, the CRC cell lines HCT8, HCT15, HCTI16, HT29, and SW480 were obtained from the American Type Culture Collection (ATCC). These cell lines underwent meticulous cultivation in DMEM medium, which was supplemented with 10% fetal bovine serum (Gibco, Carlsbad, CA, USA), 100 U/mL of penicillin, and 100 µg/mL of streptomycin. The cells were meticulously maintained at a consistent temperature of 37°C in an atmosphere enriched with 5% CO_2_. Subsequently, cellular detachment was achieved using 0.25% trypsin when cell confluency reached the range of 80-90%. All cells were assured to be free of mycoplasma contamination and had been identified pass STR profiling before starting the experiments.

### SiRNA and transfection

To transiently inhibit PSAT1 expression, Lipofectamine 3000 was used to transfect siRNA (Invitrogen, Carlsbad, CA, USA) into HCT116 and SW480 cells. Stable cell lines were established using PSAT1 siRNA knockdown (Anhui General Biology Co. LTD, Chuzhou, China). The siRNA sequence (si-PSAT1#1: 5'-CCCUAAACUUGGGAGUUAU-3'; si-PSAT1#2: 5'-ACTCAGTGTTGTTAGAGAT-3'). HCT116 and SW480 cells were transfected with 50 nmol/L siRNA according to the liposome delivery method. All experimental transfection procedure steps were inaccordance with the regulations and standards and the experiments were performed according to the instructions of the reagent manufacturer company.

### Database (DB) analysis

The exploration of the link between PSAT1 and the epithelial-mesenchymal transition (EMT) in CRC entailed the analysis of data sourced from The Cancer Genome Atlas (TCGA) database, which is accessible at the following URL: https://www.cancer.gov/about-nci/organization/ccg/research/structural-genomics/tcga. Specifically, we conducted an analysis on the normal data within the TCGA database, focusing on the correlation between PSAT1 and key EMT markers, namely E-cadherin and α-SMA, using TCGA-COAD data in the context of colorectal cancer. Additionally, for the assessment of PSAT1's impact on the prognosis of CRC patients, we availed ourselves of the Kaplan-Meier Plotter database, which is accessible at http://kmplot.com/analysis/index.php?p=background. This enabled us to investigate the relationship between PSAT1 expression levels and various clinical outcomes, including Recurrence-Free Survival (RFS) in individuals afflicted with colorectal cancer.

### Immunohistochemistry (IHC) Assay

Immunohistochemistry (IHC) was conducted in accordance with the protocols outlined in our study to evaluate the expression levels of the target proteins. Initially, sections of CRC tumor samples and adjacent normal tissue were prepared, fixed, and subsequently subjected to blocking procedures. Subsequently, the blocked tissue sections underwent an incubation step with a diluted PSAT1 antibody (catalog number: 10501-1-AP; Proteintech) for a duration of 12 hours at a temperature of 4 °C. Following this incubation, the slides were thoroughly rinsed with phosphate-buffered saline with Tween 20 (PBST) three times, with each rinse lasting for a duration of 10 minutes. After the rinsing steps, the tissue sections were exposed to secondary antibodies labeled with horseradish peroxidase (HRP), which were procured from Vector Laboratories (BA-1000, San Francisco, USA), for a duration of 90 minutes. Finally, the slides were subjected to staining utilizing DAB (Vector Laboratories, SK-4100, San Francisco, USA), followed by subsequent hematoxylin staining. The slides were sealed using neutral gum, and after the gum had dried, the images were meticulously observed under a microscope and captured for further analysis.

### Immunofluorescence (IF) Assay

Cells were cultured on coverslips (14 mm) in 24-well plates at 37°C with 5% CO_2_. After 24h, the supernatant was removed and PBS was used to wash it. Then, add 1ml 4% formaldehyde to each well for 15 min. After being washed by PBS, cells were permeabilized with 0.3% Triton X-100 at room temperature for 10 min. Then, blocked with 5% BSA for 30 min and washed by PBS three times. After that, the washed cells were incubated with polyclonal antibodies against PSAT1 (10501-1-AP; Proteintech, 1: 50), overnight at 4°C in darkness. The slides were washed with PBST for 3 times, and the excess liquid on the slides was blotted dry with absorbent paper. The diluted 488 fluorescent secondary antibody was added to the slides and incubated in a wet box for 10min at 37 °C at dark. DAPI (Biosharp Biotech, Hefei, China, 1: 1000) was used to counterstain the nuclei for 10 min. The slides were mounted on coverslips with an anti-fade mounting medium. All the images were collected by an upright fluorescence microscope (Olympus BX51, Japan). Each experiment was repeated 3 times.

### Western blot

The Western blotting and subsequent protein visualization procedures were conducted in accordance with established methodologies outlined in relevant literature sources 44,45. The primary antibodies employed in this study were as follows: Anti-PSAT1 (#10501-1-AP, Proteintech, Wuhan, China), Anti-E-cadherin (#20874-1-AP, Proteintech, Wuhan, China), Anti-α-SMA (#19245T, CST, Boston, USA), Anti-Cyclin D1 (#26939-1-AP, Proteintech, Wuhan, China), Anti-P21 (#10355-1-AP, Proteintech, Wuhan, China), Anti-Phospho-AKT (#28731-1-AP, Proteintech, Wuhan, China), Anti-AKT (#10176-2-AP, Proteintech, Wuhan, China), Anti-Phospho-P38 (#28796-1-AP, Proteintech, Wuhan, China), Anti-P38 (#14064-1-AP, Proteintech, Wuhan, China), Anti-Phospho-P65 (#3033T, CST, Boston, USA), Anti-P65 (#8242T, CST, Boston, USA), Anti-GAPDH (#60004-1-lg, Proteintech, Wuhan, China), β-Tubulin (#66031-1-Ig, Proteintech, Wuhan, China), β-actin (#66009-1-lg, Proteintech, Wuhan, China). These primary antibodies were employed in conjunction with appropriate secondary antibodies obtained from Cell Signaling Technology (#7076 or #7074, Boston, USA) and applied to the membrane at room temperature for a duration of 1 hour. Finally, electrochemiluminescence (ECL) reagent sourced from Beyotime (Shanghai, China) was utilized for visualization and detection of protein bands. The resultant data were subjected to analysis using Image J software (version 1.8.0, National Institutes of Health, Bethesda, USA).

### RNA isolation and quantitative Real-Time PCR (qRT-PCR)

In the course of this experiment, total cellular RNA was extracted employing the Trizol method, utilizing reagents from Biosharp, Beijing, China. Subsequently, we carried out cDNA synthesis utilizing the SYBR Premix EX Taq™ kit (Takala, Dalian, China). Real-time quantitative PCR (qPCR) was executed using the SYBR Premix EX Taq™ kit (Takala, Dalian, China). The described procedure was carried out utilizing the ABI 7500 real-time PCR system, manufactured by Applied Biosystems in Foster City, USA.The primer sequences utilized for the amplification of target genes were as follows: PSAT1 Sequence: 5'-GGGTAGGTCCCGTCTACTCC-3' (forward) and 5'-CCAAAGCCAATTCCATTCAC-3' (reverse). E-cadherin Sequence: 5'-TACACTGCCCAGGAGCCAGA-3' (forward) and 5'-TGGCACCAGTGTCCGGATTA-3' (reverse). α-SMA Sequence: 5'-CAGGATGCAGAAGGAGATCA-3' (forward) and 5'-TCCACATCTGCTGGAAGGTA-3' (reverse). GAPDH Sequence: 5'-GCACCGTCAAGGCTGAGAAC-3' (forward) and 5'-TGGTGAAGACGCCAGTGGA-3' (reverse). PCR primers were designed and formulated by Universal Biology (Anhui General Biology Co. LTD, Chuzhou, China). The GAPDH was used as an endogenous control. Relative gene expression was determined by comparing the 2-ΔΔCT method.

### Migration and invasion assays

Transwell assay can test CRC cells migration and invasion. Briefly, colorectal cancer cells (1.5×10^4^ cells) were resuspended in 200μl DMEM medium without FBS and dropped to the top of each transwell chamber using a pipettor. And medium supplemented with 10% FBS was also added inside the 24-well plate and in the lower part of the chamber. After the CRC cells were cultivated at 37℃ in a 5% CO_2_ humidified environment for 48h, they were fixed and stained. Five sites were randomly selected and cell numbers were counted under a microscope. Cell invasion assays were performed using similar experimental methods as described above. The difference is that matrigel was coated in the chamber above the transwell chamber (BD Biosciences, San Jose, CA, USA) for 6 hours before cell seeding. Then after 48 h of cell culture, the subsequent invasion assay was performed using a similar protocol as the migration assay.

### Wound healing assays

The CRC cells (3×10^5^) were counted in 6-well plates for culture. After 16 h, the 10% serum complete medium in the six-well plate was replaced with fresh medium containing 1% of low concentration serum. A uniformly shaped wound scratch was made on each well with a 10μl pipette tip after the cells had reached 90% confluence, according to technical methods described elsewhere in the literature. To remove dead loose cells, let the scratch assay result better. We used PBS to gently wash the cells twice and more to and add serum-free medium to continue the culture. In order to ensure the comparability of the wounds of the same wound, multiple positioning marks were made in the center of the exposed surface. The scratch area was photographed by inverted microscope at 0 and 48 h. Image J software was used to analyze the scratch test results and detect the migration ability of CRC cells. Experiments and data need to be repeated three or more times.

### CCK-8 viability assays

To assess the proliferation capacity of CRC cells, we employed the CCK-8 assay, utilizing reagents obtained from Biosharp in Beijing, China. Initially, CRC cells were seeded in 96-well plates, with each well containing 100 μl of medium, at a density of 3×10^3^ cells. These cells were then divided into five distinct groups and cultured for various durations: 0 hours, 24 hours, 48 hours, 72 hours, and 96 hours.Following the respective incubation periods, the cells were treated with a 10% CCK-8 solution and maintained at a temperature of 37°C for a duration of 2 hours. Subsequently, we measured the absorbance (a) value at a wavelength of 450 nm and calculated cell viability by determining the ratio of the absorbance (OD) value relative to the control group.

### Clone formation assay

A total of 1×10^3^ cells were cultured in a culture dish, which was placed in an incubator at 37℃ with a CO_2_ concentration of 5% for 7 to 14 days (the specific time was mainly determined according to the colony-forming ability of each cell). After appropriate cell density was observed, the medium in the culture dish was then decanted and washed twice with PBS, the cells were fixed with methanol for 30 min, then stained with 0.5% crystal violet for 30 min, and finally washed and dried with PBS. Finally, photographs were taken and recorded, and then more than five different fields of view were selected for counting and statistics. The data were repeated three times.

### Drug treatment

LY294002 (Shanghai Biyuntian Biotechnology Co., LTD, Shanghai, China) wasdissolved in DMSO. The drug dose was selected as the experimental dose (LY294002 as an example, 10μM). The above drug concentrations met the experimental requirements and were used in all cell drug experiments in this paper. In this study, HCT116 and SW480 cells were treated with LY294002 alone or in combination (at the same time) for 24 hours. As a control group, only 0.01% DMSO was added. DMSO concentrations were the same in all variants, both control group and experimental group.

### Statistical analysis

Each individual experiment was meticulously repeated a minimum of three times, and often more, to ensure the reliability and robustness of our findings. The collected data from these experiments were subsequently subjected to thorough analysis using GraphPad Prism 7.0 software. The results derived from each independent experiment were presented in the form of mean values accompanied by their corresponding standard deviations (mean±SD). To compare means between two experimental groups, we employed a two-sided t-test, and statistical significance was determined using a threshold of P < 0.05. Additionally, in situations where multiple experimental groups were involved, we utilized one-way analysis of variance (ANOVA) in conjunction with the Dunnett test to evaluate and compare means across the various groups. Once more, statistical significance was established at a significance level of P < 0.05.

## Results

### PSAT1 was down-regulated in CRC and positively associated with poor prognosis of CRC

**Figure 1:**
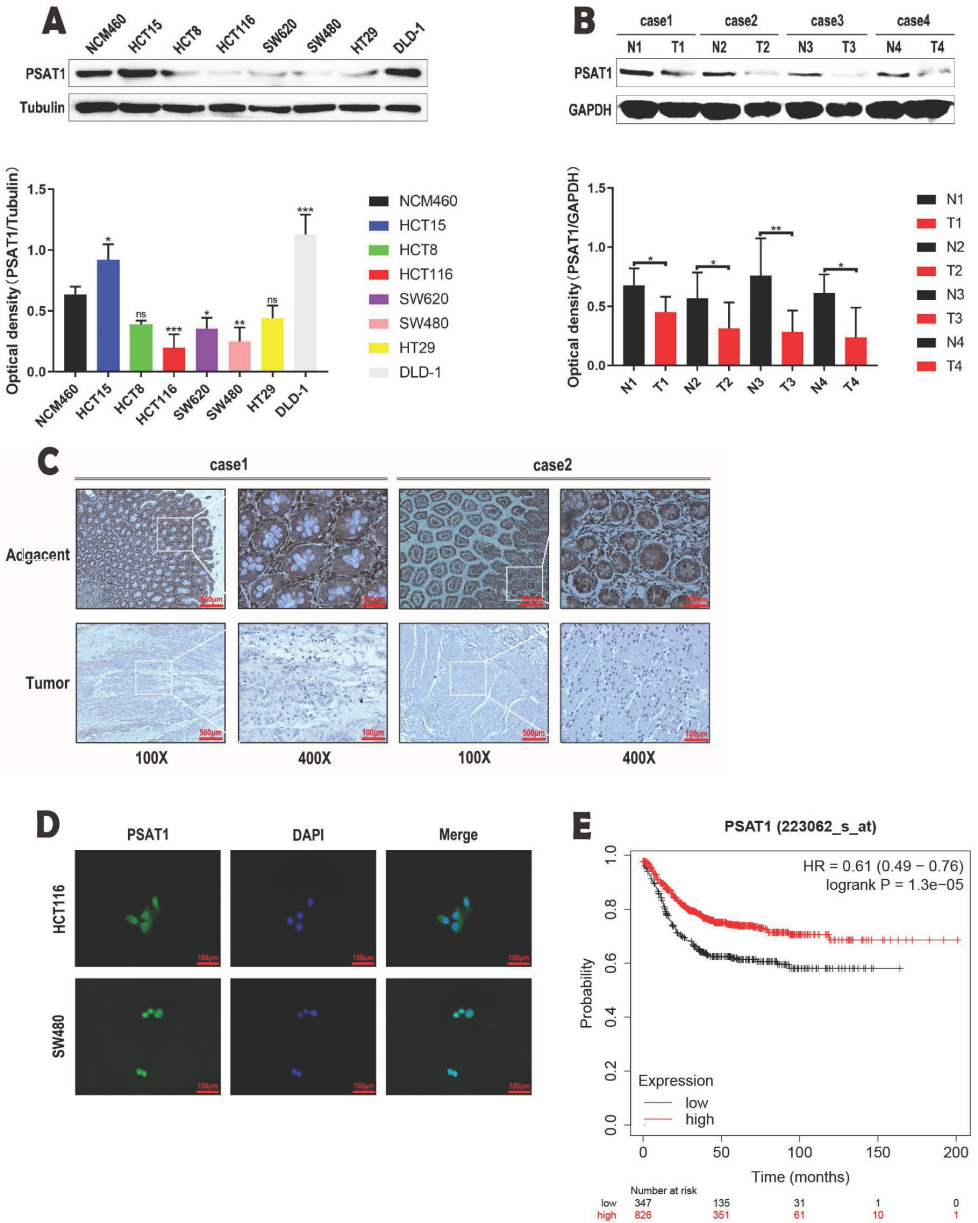
** Expression of PSAT1 in colorectal cancer cells.** (A)It shows PSAT1 protein expression levels in colorectal cancer cells as determined by Western blot analysis. (B)It shows PSAT1 protein expression levels in colorectal cancer tissues and adjacent tissues as determined by Western blot analysis. (C)It presents representative images of PSAT1 immunohistochemistry (IHC) staining in CRC tissues and adjacent normal tissues. (D)The localization of PSAT1 was detected by immunofluorescence staining. (E)It displays Kaplan-Meier overall survival curves of 1173 CRC patients stratified by low and high PSAT1 levels.The data was presented as mean ± SD of three independent experiments. *P < 0.05, **P < 0.01, ***P < 0.001, ****P < 0.0001.

Our study was designed to elucidate the expression patterns of PSAT1 in CRC cells. The results from Western blot analysis yielded noteworthy findings, indicating that the protein expression levels of PSAT1 were notably lower in various colorectal cancer cell lines (HCT8, HCT116, SW620, SW480, and HT29) when compared to human normal colonic epithelial cells (NCM460) (Figure 1A). These collective findings suggest that PSAT1 tends to exhibit a relatively low expression profile in the majority of colorectal cancer cell lines. Furthermore, in our subsequent experiments, we selected HCT116 and SW480 cells due to their representation as well-established colorectal cancer cell lines. Interestingly, the protein expression levels of PSAT1 in HCT116 and SW480 cells were significantly lower in comparison to other CRC cell lines. Therefore, we inferred that PSAT1 might be a tumor suppressor gene. In order to test this hypothesis, we selected the patient's colorectal cancer tissues and adjacent tissues for WB experiments. We found that PSAT1 expression was lower in CRC tissues and higher in adjacent normal tissues (Figure 1B). To validate these observations, we conducted IHC staining on CRC tumor samples and adjacent paracancerous tissues, which further confirmed the decreased expression of PSAT1 in CRC tissues (Figure 1C). And we found that PSAT1 was expressed in both nucleus and cytoplasm by IF localization assay (Figure 1D). Additionally, through Kaplan-Meier analysis, we uncovered a distinct correlation between PSAT1 expression and the prognosis of colorectal cancer patients. Specifically, individuals with low PSAT1 expression exhibited a less favorable prognosis, while those with high PSAT1 expression displayed a more promising prognosis (Figure 1E). It is worth noting that our analysis also revealed a positive association between PSAT1 expression and Recurrence-Free Survival (RFS) in colorectal cancer patients.

### Knockdown of PSAT1 can promote the migration, invasion, proliferation and colony formation of cancer cells

**Figure 2:**
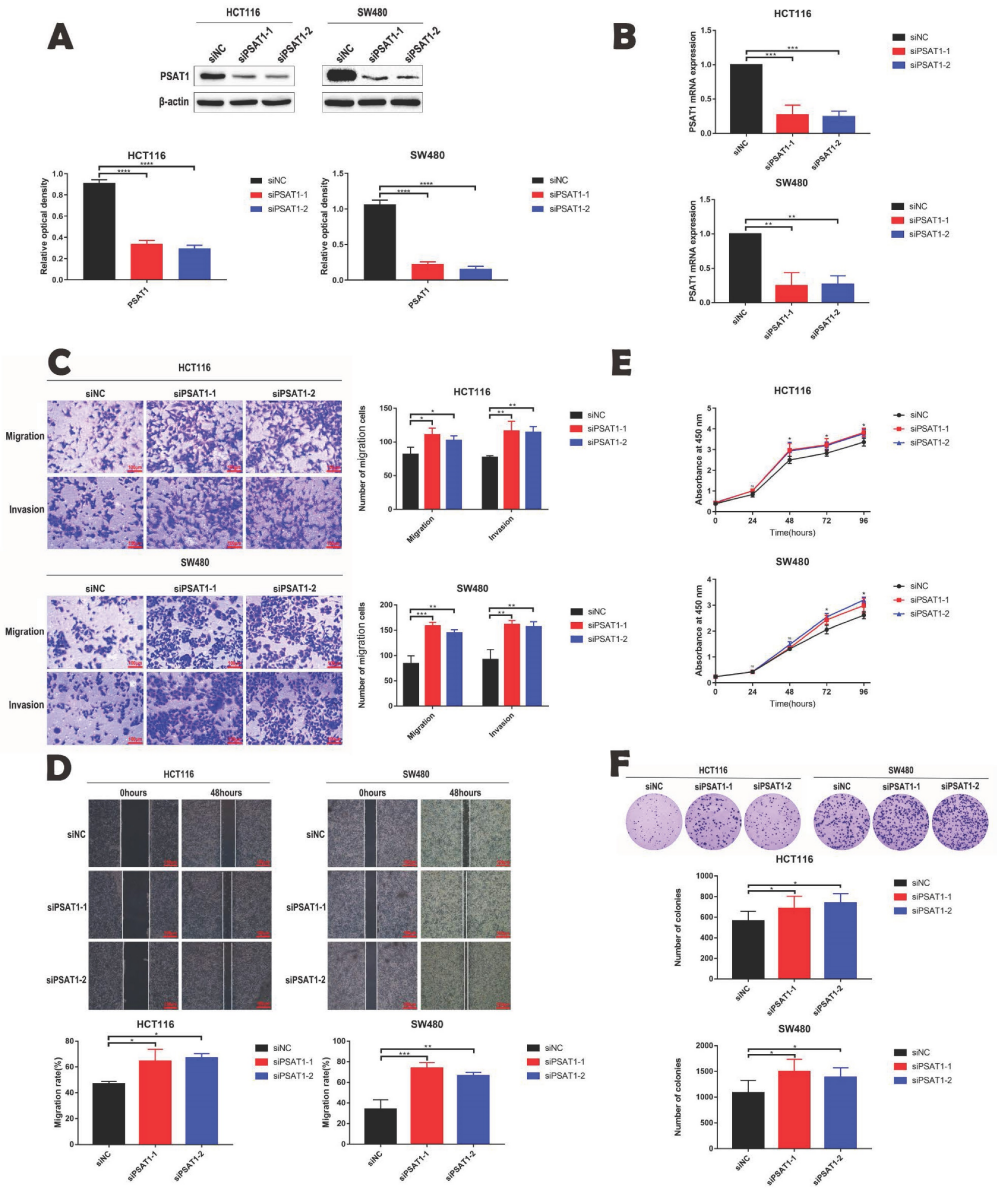
** PSAT1 inhibits the development of malignant phenotypes in colorectal cancer cells.** (A and B) Panels demonstrate the expression of PSAT1 in HCT116 and SW480 cells transfected with PSAT1 (Small interference knock-down group) and empty vector (negative control group) using Western blot and quantitative real-time polymerase chain reaction (qRT-PCR). (C)It presents the results of Transwell assays to assess cell migration and invasion influenced by PSAT1. (D)It shows the results of wound healing assays evaluating cell migration affected by PSAT1. (E)It displays the results of the Cell Counting Kit-8 (CCK-8) assay measuring cell proliferation influenced by PSAT1. (F)It illustrates the findings of the colony formation assay assessing colony formation ability influenced by PSAT1.The data was presented as mean±SD of three independent experiments. *P < 0.05, **P < 0.01, ***P < 0.001, ****P < 0.0001. si, small interference; NC, negative control.

We conducted an investigation into the impact of PSAT1 on the malignant phenotype of HCT116 and SW480 by employing si-PSAT1 to knock down PSAT1 expression in CRC cells. Our primary objective was to explore the role of PSAT1 in the progression of CRC. To ascertain the efficiency of si-PSAT1 knockdown, we conducted qPCR and Western blotting (Figure 2A and B), which confirmed the successful reduction of PSAT1 levels. Subsequently, we performed Transwell assays, which indicated that silencing PSAT1 resulted in a weakly enhancement of the migration and invasion abilities of HCT116 and SW480 cells (Figure 2C). Moreover, the wound healing assay demonstrated that silencing PSAT1 weakly strengthened the migration capacity of these cells (Figure 2D). In addition, the CCK-8 assay revealed that silencing PSAT1 led to an increase in the proliferation of HCT116 and SW480 cells (Figure 2E). Lastly, the colony formation assay indicated that silencing PSAT1 had a modest impact on the colony formation ability of HCT116 and SW480 cells (Figure 2F). Collectively, our study outcomes unveil the influence of PSAT1 on the migration, invasion, and proliferation of CRC cells, shedding light on its role in these critical aspects of CRC progression.

### PSAT1 is involved in EMT process induced by PI3K/AKT signaling pathway

**Figure 3:**
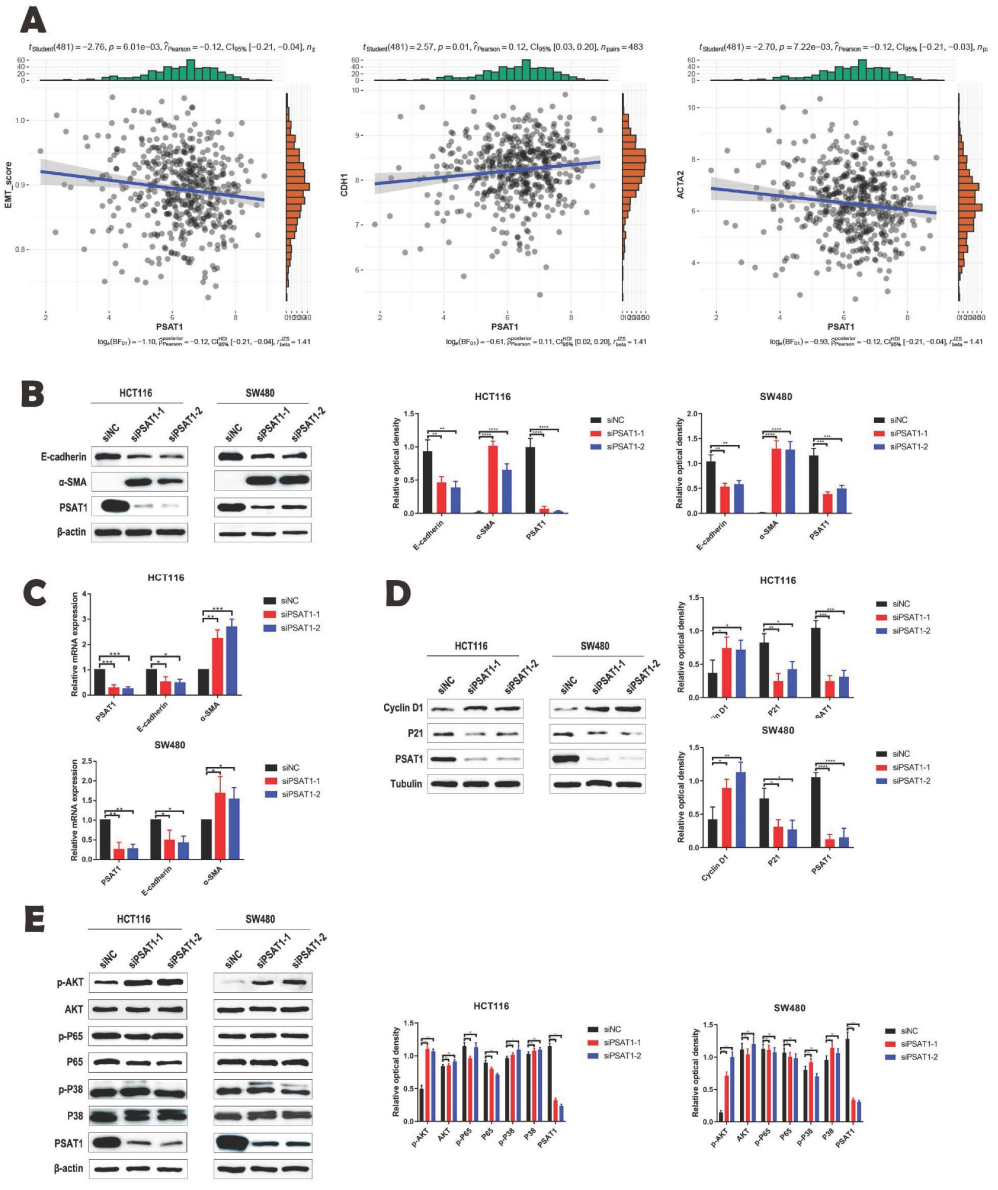
** PSAT1 is involved in EMT process induced by PI3K/AKT signaling pathway.** (A) It explores the correlation between PSAT1 and EMT marker genes in CRC. (B and C) Panels present Western blot and qRT-PCR results assessing the effect of PSAT1 on E-cadherin and α-SMA expression. (D)It displays Western blot results evaluating the effect of PSAT1 on Cyclin D1 and P21 expression. (E)It illustrates Western blot results detecting the effect of PSAT1 on p-AKT, AKT, p-P65, P65, p-P38, and P38 expression.The data was presented as mean ± SD of three independent experiments. *P < 0.05, **P < 0.01, ***P < 0.001, ****P < 0.0001. si, small interference; NC, negative control.

In our analysis utilizing the TCGA database, we employed the ssGSEA algorithm to assess PSAT1 scores for the CRC samples. These results unveiled a significant correlation between PSAT1 and key factors associated with EMT, specifically E-cadherin and α-SMA in CRC (Figure 3A). To further substantiate the influence of PSAT1 on EMT and its pivotal role in tumor metastasis, we introduced si-PSAT1 into HCT116 and SW480 cells. The subsequent results obtained through both qPCR and Western blotting (WB) demonstrated a down-regulation of the epithelial marker, E-cadherin, and an up-regulation of the mesenchymal marker, α-SMA. These findings strongly suggest that si-PSAT1 amplifies the activation of EMT in CRC (Figure 3B, C). In addition, we sought to investigate the regulatory mechanism of PSAT1 concerning the proliferation ability in CRC. To achieve this, we evaluated the impact of si-PSAT1 on key cell cycle proteins, namely Cyclin D1 and P21. The WB results revealed an up-regulation of Cyclin D1 protein and a down-regulation of P21, indicating that si-PSAT1 promotes the proliferation capacity of CRC cells (Figure 3D). For a comprehensive understanding of the molecular mechanism underlying PSAT1's role in EMT, we meticulously selected several downstream molecules within classical signaling pathways associated with EMT for experimental evaluation. However, the WB results indicated that knockdown of PSAT1 did not induce notable changes in the total expression levels of phosphorylated P65 and phosphorylated P38. Similarly, the expression levels of P65 and P38 remained relatively stable. Nevertheless, it was observed that knockdown of PSAT1 resulted in an increase in the total expression of phosphorylated AKT, with the expression level of AKT remaining largely unchanged. This outcome aligns with existing research indicating a link between the development of cancer and the PI3K/AKT signaling pathway. Thus, our results suggest that si-PSAT1 activates the PI3K/AKT signaling pathway (Figure 3E).

### LY294002 can reverse PSAT1 regulation of EMT progress in CRC

**Figure 4:**
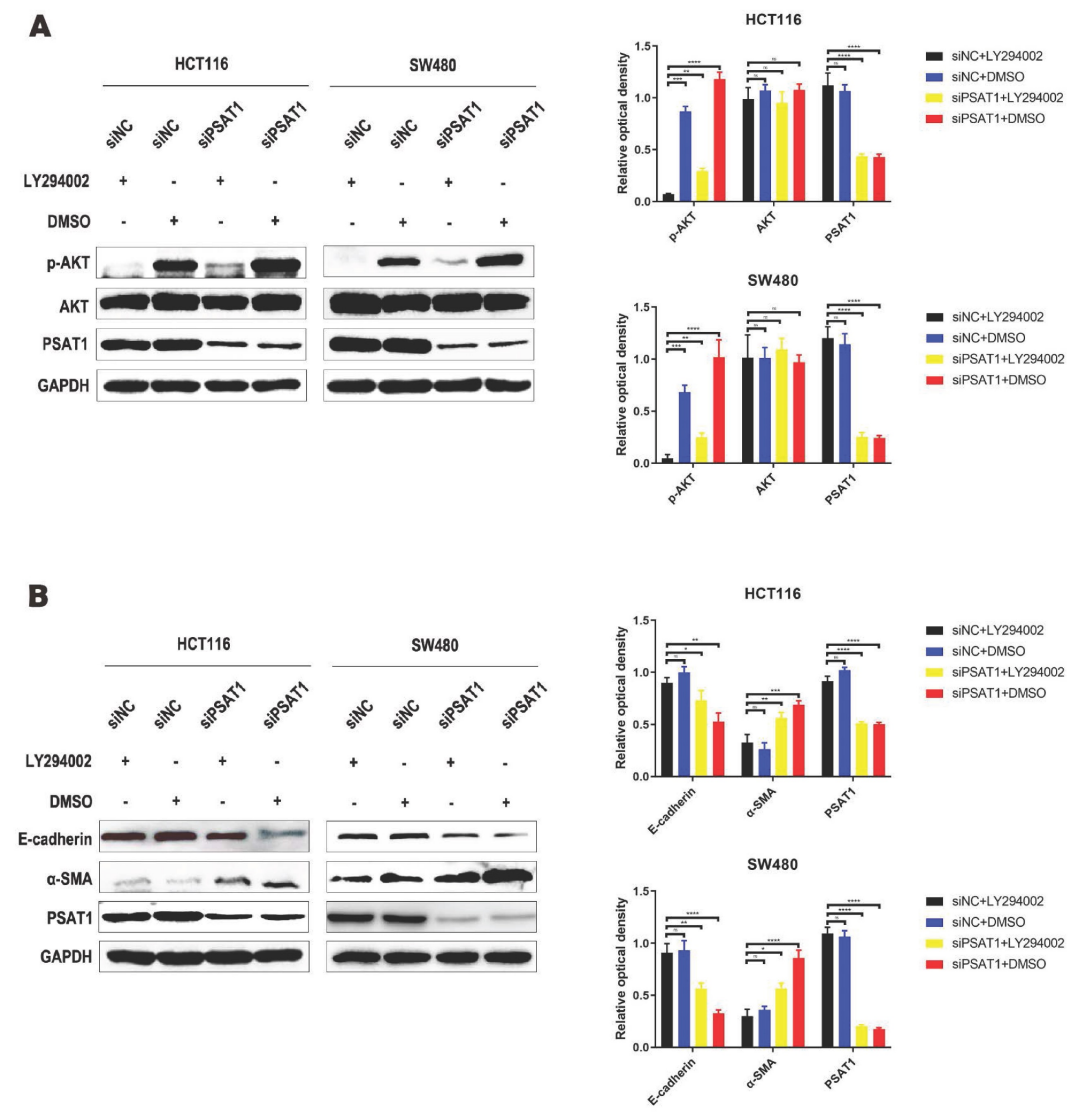
** LY294002 can reverse PSAT1 regulation of EMT progress in CRC.** (A)It depicts CRC cells treated with LY294002, and Western blot analysis assessing the expression of p-AKT, AKT, and PSAT1. (B)It presents Western blot results evaluating the expression of E-cadherin, α-SMA, and PSAT1.The data was presented as mean ± SD of three independent experiments. *P < 0.05, **P < 0.01, ***P < 0.001, ****P < 0.0001. si, small interference; NC, negative control.

To investigate whether PSAT1 contributes to EMT progression in HCT116 and SW480 cells by modulating the activation of the PI3K/AKT pathway, we conducted experiments to determine whether LY294002, a PI3K/AKT pathway inhibitor, could reverse the effects of PSAT1 on EMT. We assessed the expressions of PSAT1, E-cadherin, α-SMA, AKT, and p-AKT in SiPSAT1-transfected colorectal cancer cells treated with the PI3K/AKT signaling pathway inhibitor LY294002.Previous research has established that PSAT1 knockdown significantly elevates p-AKT expression in HCT116 and SW480 cells. However, this effect was entirely counteracted by the administration of the PI3K/AKT inhibitor LY294002. Interestingly, the expression levels of AKT and PSAT1 remained unaffected by the inhibitor, showing minimal changes in their expression, in line with our earlier observations. Consequently, these results further emphasize the potential interplay between PSAT1 and the PI3K/AKT signaling pathway (Figure 4A). In terms of EMT-related markers, the knockdown of PSAT1 led to a reduction in the expression of E-cadherin and an increase in the expression of α-SMA in HCT116 and SW480 cells. However, subsequent intervention with LY294002 resulted in the upregulation of E-cadherin and the downregulation of α-SMA. Notably, the expression of PSAT1 remained consistent with previous findings (Figure 4B). Taken together, these findings signify that the PI3K/AKT inhibitor LY294002 has the capability to restore the protein expression levels of p-AKT, E-cadherin, and α-SMA in siPSAT1-transfected cells, suggesting a potential regulatory role of the PI3K/AKT pathway in the context of PSAT1-mediated EMT.

### The PI3K/AKT Inhibitor LY294002 rescued the function of PSAT1

**Figure 5:**
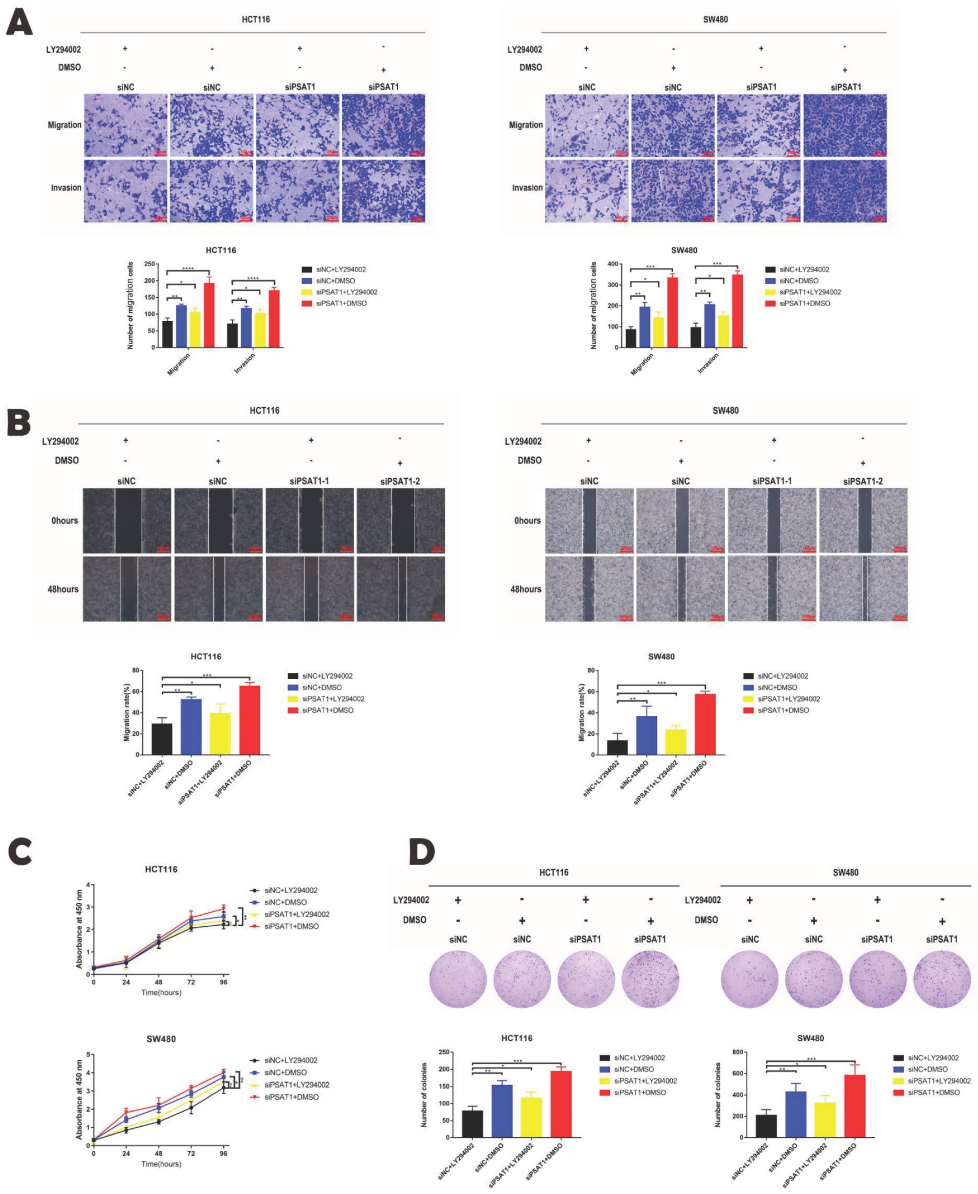
** LY294002 reversed the effect of PSAT1 on the phenotype of CRC cells.** (A)It displays the results of the Transwell assay assessing the migration and invasion of HCT116 and SW480 cells treated with LY294002. (B)It shows the results of the wound healing assay evaluating the migration abilities of HCT116 and SW480 cells treated with LY294002. (C)It presents the results of the CCK-8 assay measuring cell proliferation in response to LY294002 treatment. (D)It illustrates the findings of the colony formation assay assessing colony formation ability upon LY294002 treatment.The data was presented as mean ± SD of three independent experiments.*P < 0.05, **P < 0.01, ***P < 0.001,****P < 0.0001. si, small interference; NC, negative control.

**Figure 6:**
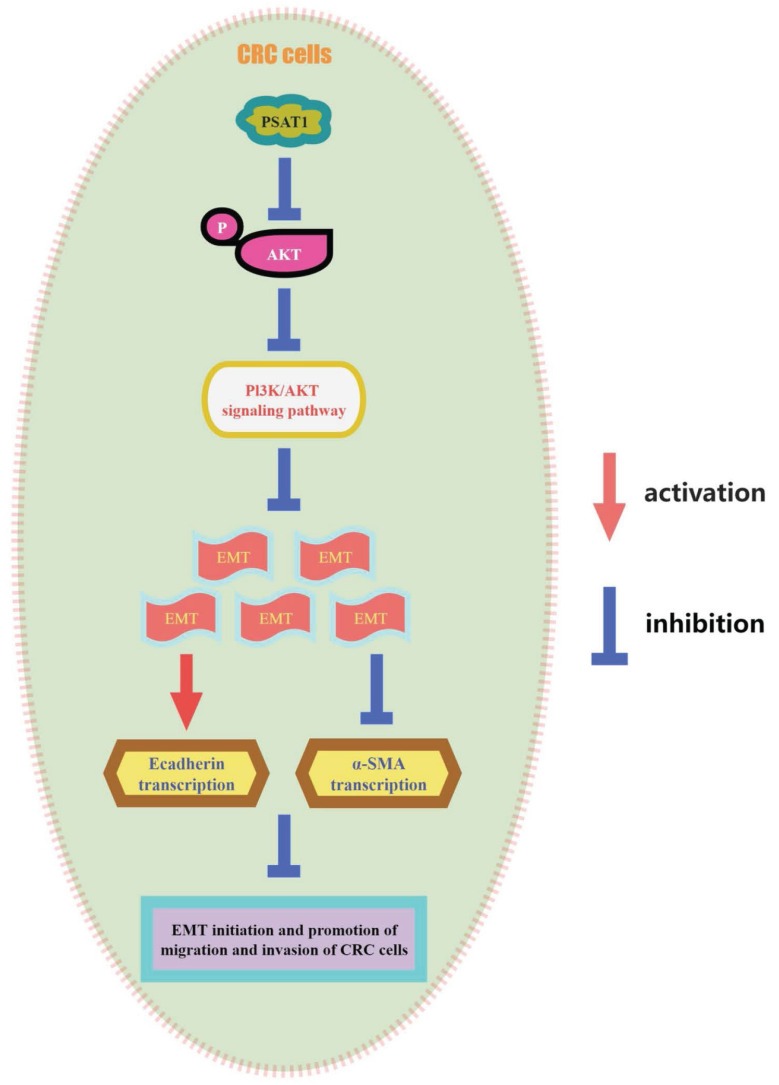
** Schematic model of the underlying molecular mechanism of PSAT1 on CRC metastasis.**This schematic model summarizes the underlying molecular mechanism of PSAT1 in CRC metastasis. PSAT1 inhibits the migration and invasion of colorectal cancer cells by attenuating the phosphorylation of AKT and suppressing the PI3K/AKT pathway, thereby impeding the progression of EMT.

Our previous phenotypic experiments yielded valuable insights, demonstrating that the knockdown of PSAT1 in HCT116 and SW480 cells promoted cell migration, invasion, and proliferation. However, intriguingly, we observed that this effect was completely reversed by the administration of the PI3K/AKT inhibitor LY294002. Upon using the inhibitor, it was evident that the cell migration and invasion abilities of the PSAT1-knockdown group were significantly inhibited (Figure 5A and B). Additionally, PSAT1-siRNA had a modest stimulatory effect on the proliferation of CRC cells, which was subsequently suppressed by the PI3K/AKT inhibitor LY294002 (Figure 5C). These trends were further corroborated by clonogenic assays (Figure 5D). Consequently, these compelling results provide strong evidence that the PI3K/AKT inhibitor LY294002 can effectively reverse the functional effects exerted by PSAT1 in colorectal cancer cells. This observation solidifies our earlier conclusion that PSAT1 mediates EMT through the PI3K/AKT signaling pathway in CRC (Figure 6).

## Discussion

PSAT1, an enzyme involved in serine synthesis, has garnered substantial attention in recent years due to its pivotal role in cancer progression, particularly in relation to invasion and metastasis, a critical aspect in clinical oncology 46,47. In our current investigation, we have identified that PSAT1 exhibits low expression in colorectal cancer cells and CRC tumor samples. Importantly, we have established its regulatory role in driving the malignant phenotype of CRC cells, and it has emerged as a marker positively associated with poor prognosis. Nevertheless, the precise biological functions of PSAT1 in CRC, notably its involvement in metastatic progression, remain insufficiently elucidated. Emerging research has provided compelling evidence of PSAT1's crucial role in the initiation and advancement of distant metastasis in breast cancer 48. Moreover, in mechanistic studies focusing on the malignant characteristics of hepatocellular carcinoma (HCC) cells, the RP4-694A7.2 lncRNA has been shown to promote cell proliferation and metastasis through its interaction with PSAT1 49. Notably, ectopic expression of MEG3 has demonstrated the ability to significantly inhibit the proliferation, migration, invasion, and EMT of esophageal squamous cell carcinoma (ESCC) cells by down-regulating PSAT1 expression 50. In the context of colorectal cancer, it is widely acknowledged that metastasis remains a major challenge in the effective treatment of malignant progression 51. EMT, a pivotal phenotypic transition, plays a central role in tumor progression and metastasis in colorectal cancer 52. In light of these findings, the exploration of the relationship between PSAT1 and EMT holds significant promise and may offer novel insights for the clinical management of colorectal cancer.

In various tumor types, the loss of E-cadherin expression, a marker protein associated with epithelial origin, and the induction of markers such as N-cadherin, Vimentin, and α-SMA, typically linked with stromal cells, play pivotal roles as indicators of the EMT process 53. In CRC, the occurrence of EMT is characterized by distinct features, including the loss of apical-basal polarity, disruption of cell-cell junctions, and the downregulation of epithelial markers, notably E-cadherin. Conversely, the induction of a mesenchymal phenotype and the expression of related markers such as N-cadherin or α-SMA contribute to altered cellular behavior, resulting in increased cell migration and invasion capabilities 54.

In our current study, through an analysis of the TCGA database, we have discerned a negative correlation between PSAT1 and EMT. Specifically, PSAT1 expression was inversely associated with EMT, reinforcing our hypothesis that PSAT1 plays a role in EMT in CRC. Further experimentation involving the knockdown of PSAT1 in colorectal cancer cells revealed a down-regulation of E-cadherin expression and an upregulation of α-SMA expression. Additionally, the knockdown of PSAT1 was found to promote the migration and invasion of CRC cells. In summary, these findings offer compelling evidence supporting the role of PSAT1 in driving the process of EMT in the context of CRC. Consequently, obtaining a thorough comprehension of the underlying molecular mechanisms governing EMT becomes essential for the advancement of treatment strategies and the enhancement of prognosis for CRC patients. In recent years, with the continuous refinement of scientific research and technological advancements, extensive investigations into the molecular mechanisms of EMT have been conducted, revealing a plethora of signaling pathways that can induce EMT 55. Notably, the PI3K/AKT signaling pathway has been identified as a mediator of EMT development in colorectal cancer 56. Furthermore, it is noteworthy that PSAT1 has been documented to stimulate cell proliferation while inhibiting apoptosis through the activation of the PI3K/AKT signaling pathway, thereby imparting increased resistance to cisplatin in cervical cancer cells 57.

Similarly, PCSK9 has been demonstrated to augment the proliferation, migration, and invasion of colon cancer cells *in vitro* by inducing EMT and activating the PI3K/AKT signaling pathway 58. Moreover, reports have highlighted that EMT can be initiated by the circEPSTI1/miR-942-5p/LTBP2 axis in oral squamous cell carcinoma (OSCC) cells. This axis not only fosters cell proliferation, migration, and invasion *in vitro* but also *in vivo*, primarily through the mediation of the PI3K/AKT/mTOR signaling pathway 59. These findings underscore the multifaceted roles of various molecular pathways in modulating critical aspects of cancer progression.

In our investigation, we noted that the knockdown of PSAT1 resulted in an elevation in the overall expression of phosphorylated AKT, thereby furnishing compelling evidence that si-PSAT1 indeed triggered the activation of the PI3K/AKT signaling pathway. This observation reinforces the complex interrelationship between PSAT1 and the PI3K/AKT pathway, offering valuable insights into a potential mechanism by which PSAT1 governs EMT in the context of CRC 60. These findings contribute to our understanding of the intricate molecular processes underlying EMT regulation in colorectal cancer.

LY294002 is a well-established and widely recognized inhibitor of the classical PI3K/AKT signaling pathway, and it has found extensive utility in clinical applications. Our study unequivocally demonstrates that LY294002 effectively reverses the siPSAT1-induced EMT process and the expression of p-AKT, thereby affecting the malignant phenotype of colorectal cancer cells. These findings provide compelling evidence of a significant correlation between the PI3K/AKT signaling pathway and EMT in colorectal cancer.

In summary, our study has unveiled the substantial impact of PSAT1 on the invasion and migration of colorectal cancer cells, shedding light on its role in regulating the EMT process through its interaction with the PI3K/AKT signaling pathway. This intricate interplay ultimately influences the progression of colorectal cancer, highlighting the potential of PSAT1 as a promising therapeutic target for the treatment of CRC. Importantly, the knockdown of PSAT1 was shown to activate the expression of p-AKT, upregulate α-SMA, and downregulate E-cadherin, resulting in enhanced cell migration, invasion, and proliferation capacities, primarily achieved through the modulation of EMT and the PI3K/AKT signaling pathway. Targeting PSAT1 to regulate the occurrence, proliferation, invasion, and EMT process of colorectal cancer represents a novel therapeutic approach. Subsequent studies may explore strategies for controlling the migration, invasion, and proliferation of colorectal cancer by manipulating EMT through PSAT1 and its potential combination with other therapeutic agents, offering a fresh perspective on combating the malignant phenotype of colorectal cancer.

## Conclusion

Through this research, we found that PSAT1 has relationship with EMT in CRC cells. The PSAT1 prevents the development of EMT by inhibiting the PI3K/AKT pathway in CRC. Our analysis not only enhances the understanding of PSAT1 and cell metastasis but also indicates its potential signaling pathways in CRC cells.
